# Examining the Impact of Long-Term Care Insurance on the Care Burden and Labor Market Participation of Informal Carers: A Quasi-Experimental Study in China

**DOI:** 10.1093/geronb/gbae023

**Published:** 2024-02-23

**Authors:** Xingtong Pei, Wei Yang, Mingming Xu

**Affiliations:** School of Public Health (Shenzhen), Sun Yat-sen University, Shenzhen, China; Department of Global Health and Social Medicine, Faculty of Social Science and Public Policy, King’s College London, London, UK; School of Public Health (Shenzhen), Sun Yat-sen University, Shenzhen, China; (Social Sciences Section)

**Keywords:** China, Informal carer, Labor market participation, Long-term care insurance, Staggered difference-in-differences

## Abstract

**Objectives:**

Existing evidence from high-income countries suggests that policies aimed at enhancing access to formal care can reduce the burden on informal carers and facilitate their reentry into the labor market. However, there is limited evidence regarding the specific carers who have been most affected by such insurance. This study focuses on China’s long-term care insurance (LTCI) and examines its effects on informal care burden and the labor market participation of different types of informal carers.

**Methods:**

Drawing data from the China Health and Retirement Longitudinal Study of 2011, 2013, 2015, and 2018, we employ a staggered difference-in-differences (DID) model with propensity score matching to analyze the impact of LTCI. To explore time-varying DID estimates, we adopted the DID event study design.

**Results:**

Our study demonstrates that LTCI substantially alleviates the burden on informal carers while markedly boosting labor market participation. Notably, we found a more pronounced decrease in care burden among spouses, amounting to a reduction of 8.5 hr per month. Concurrently, LTCI’s impact on enhancing labor market participation was more significant among younger household members, reflected in an average income increase of 4,534 yuan per year. Furthermore, subgroup analysis highlights that LTCI primarily benefits informal carers providing care for older people with low income or those who were farmers or previously engaged in informal sectors.

**Discussion:**

Our study demonstrates that LTCI has led to a reduction in care burdens and an enhancement in labor market participation. The impact is especially pronounced for informal carers of older people with low income or those with backgrounds in farming or informal work sectors.

In OECD countries, the proportion of people aged older than 65 in the total population was over 17% in 2019, and this figure is predicted to reach 26.7% by 2050 (OECD, 2021). As individuals age, they often encounter physical and cognitive limitations that necessitate assistance with daily activities. This reliance on support from family, friends, or others, provided without formal agreements or monetary compensation, is commonly known as informal care (Yang, Wu, et al., 2021). Informal care involves aiding individuals in need due to illness, disability, or related conditions.

Policies aimed at improving access to formal long-term care (LTC) frequently result in indirect positive effects on informal carers, including the reduction of their care burden and enhancement of labor market participation. Publicly funded long-term care insurance (LTCI) has been implemented in various high-income countries over the past few decades and is increasingly gaining popularity in middle-income countries (Chen et al., 2020). Research suggests that these insurance programs often provide broader formal coverage against LTC risks. This contribution can lead to a reduction in the reliance on informal care by improving the affordability of formal care (Bonsang, 2009), easing the substantial challenges faced by family members (Ettner, 1994), and facilitating the reentry of individuals into the job market (Fu et al., 2017). Despite evidence indicating potential benefits of LTCI for informal carers, additional research is needed to identify the specific caregiver groups experiencing the most significant impact on their care burden and labor market participation due to LTCI.

China is among the pioneering low- and middle-income countries (LMICs) to launch a publicly funded LTCI program, and the achievements thus far have been remarkable (Chang et al., 2020). In 2016, the Chinese government launched the first LTCI pilot program in 15 cities and two key provinces, which expanded to an additional 14 cities in 2020. The program aimed to offer financial support and coverage for LTC services, particularly for older people and those with functional limitations or disabilities. Existing research has highlighted the program’s positive outcomes, including reduced hospitalization utilization and medical expenditures (Feng et al., 2020), improved overall health status among older people, more equitable healthcare utilization across different age groups (Liu & Hu, 2022; Ma et al., 2019), and positive influence on caregiving intensity and female labor market participation in rural areas (Lei et al., 2022). However, the impact of China’s LTCI on the care burden and labor market participation of informal carers has not been extensively studied, and few studies have examined the effects among different populations, particularly younger caregivers, such as children and grandchildren. It is also unclear whether the effects of the LTCI vary across older people from different socioeconomic status. The extent to which vulnerable groups, including low-income individuals, may benefit from LTCI also requires further investigation.

Based on the discussion above, this article adopts a quasi-experimental approach leveraging the rollout of the initial LTCI pilots in China. The study aims to investigate whether the introduction of LTCI can alleviate burdens among informal carers and promote labor market participation, especially among younger family caregivers. Subgroup analyses are conducted to examine the heterogeneous effects based on health insurance types and equivalent income. The results of this study have significant policy implications for governments in other LMICs, as well as countries with similar health and LTC systems. Data are drawn from China Health and Retirement Longitudinal Survey (CHARLS) 2011, 2013, 2015, and 2018.

## Literature Review

### Informal Care Provision and Labor Market Participation

Informal care has significant implications on the labor market participation of caregivers (OECD, 2021). Many informal carers belong to the working-age population, and they face challenges in balancing caregiving responsibilities with work obligations. Providing informal care can significantly impact labor market participation through various channels.

Firstly, the conflict between informal care provision and paid work often leads to a reduction in work hours, adjustments in work arrangements, or even the decision to exit the labor market altogether (Leigh, 2010). Providing care for someone with disabilities can be time-consuming, leaving little room for other commitments (Ho et al., 2005; Houtven et al., 2013; Longacre et al., 2017). Studies consistently show that caregiving duties are frequently cited as the primary reason for leaving the labor market (Bobinac et al., 2010; Longacre et al., 2017).

Secondly, the unpredictable nature of caregiving can result in unplanned absences from paid work, such as emergencies or accidents involving the care recipient (Leigh, 2010). These care-related absences not only lead to income loss but may also jeopardize job security (Arksey, 2002). The interruption of work can also have negative consequences on the accumulation of human capital, resulting in missed promotion opportunities and a disadvantaged bargaining position, ultimately affecting future wages and diminishing long-term labor supply.

Thirdly, the decision of informal carers to reduce their working hours or exit the labor market can have lasting consequences. Labor market frictions may make it challenging for them to reenter employment opportunities once their caregiving responsibilities have ended (Brandt et al., 2022; Tamiya et al., 2011). This challenge is particularly pronounced for informal carers aged 50 and older, as reentering the labor market becomes increasingly difficult after an extended absence.

Lastly, the burdens of informal care and the challenges of balancing work and family responsibilities have significant adverse effects on the physical and mental health of caregivers (van den Berg et al., 2004). The demanding nature of caregiving, coupled with the emotional pressure it entails, can pose obstacles for caregivers to focus on work and effectively maintain paid employment (Lee et al., 2015; Longacre et al., 2017; Weinberger et al., 1993). Such circumstances may force caregivers to leave the labor market or work part-time (Smith et al., 2020).

It is important to highlight that the impact of providing informal care on the labor market participation of informal carers can vary significantly depending on the household’s socioeconomic status, as individuals from lower socioeconomic backgrounds might lack the financial means to access paid formal care services, leading them to rely on informal care and potentially affecting the labor market participation (Brandt et al., 2022; Quashie et al., 2022). Higher-income households tend to have more options when it comes to deciding how to balance care responsibilities, such as purchasing formal care services in the market (Henz, 2006). Evidence from studies in the United States has indicated that higher-income households are more likely to engage in economic transfers and less likely to allocate significant time transfers to care recipients (Couch et al., 1999). Similarly, multiple studies consistently demonstrate a negative correlation between the likelihood of informal carers leaving the labor market and the reported annual household income (Muurinen, 1986).

### The Impact of Long-term Care Insurance on the Care Burden and Labor Market Participation of Informal Carers

The influence of LTCI on labor force participation may vary depending on the nature of subsidies provided. Previous studies have shown that in countries like Germany and the UK, cash subsidies and pension entitlements from LTCI can reduce the opportunity cost for informal carers. This reduction has been observed to negatively affect labor force supply (Carmichael & Charles, 2003; Geyer & Korfhage, 2018). In contrast, the LTCI system in other countries like Japan offers only in-kind benefits, that is, purchasing formal care. Some evidence shows that formal care provided by the LTCI can alleviate the care burden on informal carers by partially substituting informal care (Ettner, 1994; Fu et al., 2017; Stabile et al., 2006; Tamiya et al., 2011), reducing the physical and mental stress of informal carers (Boles et al., 2004), allowing them to dedicate more time to paid work or rejoin the labor market (Stabile et al., 2006), and promoting the labor force participation (Fu et al., 2017; Sugawara & Nakamura, 2014). However, others report that despite increased in-kind benefits for the older adults under LTCI, there are no significant positive effects on labor force participation (Ando et al., 2021; Fukahori et al., 2015; Geyer & Korfhage, 2015).

In addition, evidence on which specific groups of informal carers benefit the most from LTCI in terms of promoting their labor market participation is limited. Spouses often assume primary caregiving responsibilities for their partners, which can bring them more care burden and hinder their participation in formal employment. Although LTCI can provide support by offering formal care services to alleviate the burden of caregiving, it is crucial to consider that spouses may have shared financial resources and may be less reliant on labor market participation for financial stability. Conversely, adult children and grandchildren are typically in their prime working years and may face greater financial pressures. As a result, they may benefit more from LTCI if it reduces their caregiving responsibilities, allowing them to focus on their employment. When LTCI enables older parents or grandparents to access formal care services, it can enhance the ability of adult children and grandchildren to balance their caregiving responsibilities with their employment.

Moreover, it is essential to investigate whether the effects of LTCI differ across families with diverse socioeconomic backgrounds. Lower-income families often have limited access to alternative formal care options, heavily relying on familial support. LTCI has the potential to alleviate the care burden of informal carers from these families and enable them to reenter the labor market.

## Case Study: Long-term Care Insurance in China

China’s LTCI is closely linked to its social health insurance (SHI) system, which includes the Urban Employee Basic Medical Insurance (UEBMI) for urban residents with formal employment, the Urban and Rural Resident Medical Insurance (URRMI) for urban residents without formal employment and rural residents, and Government Medical Insurance. The URRMI was formed through the merger of existing insurance schemes in 2016, leading to enhanced accessibility to healthcare services for over 95% of the Chinese population. However, as the population continues to age, there is a growing reliance on medical care as a substitute for LTC due to limited resources (The State Council, 2016).

To promote professional LTC availability, provide financial assistance to disabled older people, and alleviate the burden on informal carers, the Chinese government introduced LTCI in 15 pilot cities and two key provinces in 2016. Among these cities, Qingdao was selected as a research focus due to available data. Even before nationwide policy, Qingdao had local LTCI guidelines in place since 2012. In 2020, a second round of LTCI implementation occurred in 14 pilot cities. Although the selection of pilot sites was not random but based on the application from local authorities, the final sites have covered most provinces (National Healthcare Security Administration, 2020). Presently, LTCI financing relies primarily on existing SHI funds and subsidies without additional premiums. The LTCI coverage dimensions vary among the pilot cities. Firstly, population coverage differs, with cities like Shanghai, Qingdao, and Suzhou providing LTCI coverage to all enrollees in UEBMI and URRMI, while other cities limit coverage to UEBMI enrollees only. Coverage levels for disabled older people vary across cities, with some cities covering moderate-to-severe disabilities and others extending coverage to mild disabilities or severe dementia. Secondly, the coverage of services varies, but most pilot cities include basic LTC, healthcare, and rehabilitation training provided in hospitals, institutes, or homes in their LTCI packages. Thirdly, LTCI offers cost coverage by reimbursing a fixed proportion (around 70% on average) of LTC costs, depending on disability levels, types of care, and health insurance. These variations in coverage dimensions reflect the diverse implementation strategies and evolving nature of LTCI in the pilot cities as the program continues to refine and expand its scope. Supplementary Table 1 provides a summary of the features of the LTCI.

## Methods

### Data Source and Sample Selection

We drew data from the China Health and Retirement Longitudinal Study (CHARLS) of 2011, 2013, 2015, and 2018. CHARLS collects a nationally representative sample of middle- and old-aged residents in China, covering individual-level panel data on personal information, health status, healthcare utilization, socioeconomic status, etc. (CHARLS, 2020). In addition, we merged the longitudinal data with economic data (provincial GDP per capita) from China’s National Bureau of Statistics (National Bureau of Statistics of China, 2020b).

The participants’ LTCI status was not included in the CHARLS questionnaire until the 2018 wave. To address this, we followed the approach used by previous researchers and constructed a variable indicating eligibility for LTCI (Lei et al., 2022). Specifically, we examined government policy documents and published literature to determine the time of the pilot and what was required locally to be eligible to participate in the LTCI. We found that the eligibility rule is tightly linked to individual’s SHI status (see Supplementary Table 2 for more details about the pilot cities and the timing of policy implementation). We then defined our treatment group as those who were from eligible areas for participating in the scheme, and the control group as those from the non-pilot cities.

We finally selected 62,638 observations across four waves in the study by excluding: (1) people aged below 45 and (2) observations whose dependent variables are missing. Supplementary Table 3 presents the summary statistics of the sample.

### Variable Specifications

Our study examined two dependent variables. Firstly, we measured the burden of informal care among family caregivers by calculating the hours of unpaid care provided by all relatives/children and grandchildren/spouses in the last month. This is determined by multiplying the unpaid care days by the average daily care hours (Fan & Chen, 2011). Secondly, based on previous literature, household income provides a clearer understanding of changes in economic output in the household, and it has been frequently used as a suitable measure to indicate the status of labor market participation (Bobinac et al., 2010; Cai & Kalb, 2006; Cerrutti, 2000).We did not use the variable directly indicating individual’s job market status due to the unavailability of corresponding questions in CHARLS. We constructed household income based on the following two questions in CHARLS: (1) In the past year, did you receive any wage and bonus income, excluding your pension? (2) In the past year, did the other household members receive any wage and bonus income, excluding pension? We exclude pension as this does not relate to labor market participation. We also excluded incomes from agricultural activities as these salaries cannot accurately reflect labor market participation due to various uncertain factors such as weather and geographical location, etc. (Habib-ur-Rahman et al., 2022). Furthermore, we constructed the following two additional income variables: (1) income from older household members (the main respondents, their spouses, and their parents); and (2) income from younger household members (the main respondents’ children and grandchildren), who are the primary participants in the labor force market (National Bureau of Statistics of China, 2020a). Both income variables are trimmed at 0.05% and 99.95%.

We controlled for the following covariates that may affect the burden of informal care or the labor market participation. First, we included a set of health-related variables, such as self-perceived health status (1 = excellent, 2 = very good, 3 = good, 4 = fair, 5 = poor), the number of chronic diseases, the number of activities of daily living limitations and instrumental activities of daily living limitations (Yang & Hu, 2022). Second, we further controlled for marital status (0 = married/cohabiting, 1 = single), the number of living children, the number of total household members, the number of younger/older household members, health insurance types (1 = no health insurance, 2 = UEBMI, 3 = URRMI, 4 = others), provincial GDP per capita and the following two socioeconomic status indicators: education attainment (1 = no formal education, 2 = elementary or middle school, 3 = high school and above) and the quintile of equivalent income. Equivalent income is equal to household income divided by the square root of the household size (OECD, 2011). Birth year, gender, and other time-invariant variables are controlled for by applying individual fixed effects.

In heterogeneity analyses, we analyzed the effect of the LTCI on the subgroups by health insurance types and income levels. For economic status, we divided the sample into tertiles based on the equivalent income (low income, middle income, and high income).

### Empirical Strategies

We used fixed-effects multivariate regression by adopting a staggered difference-in-differences (DID) approach with propensity score matching (PSM) to explore the effects of LTCI on burdens among informal carers and labor market participation.

#### Staggered DID model

We adopted a DID approach to explore the causal relationship between the introduction of LTCI and burdens among informal carers/labor market participation. The settings were DID with staggered adoption because the timing of policy implementation varied across the pilots (Athey & Imbens, 2022). Enrollees of the LTCI in pilot cities are included in the treatment group (Chen & Ning, 2022; Liu & Hu, 2022). The control group is consisted of the observations in non-pilot cities (see Supplementary Table 2 for more details about the pilot cities and the timing of policy implementation). To explore time-varying DID estimates, we adopted the DID event study design. It is worth noting that there is a notable issue associated with staggered DID methodologies, especially when dealing with heterogeneous treatment effects in the conventional two-way fixed effect (TWFE) model (Goodman-Bacon, 2021). This problem, known as the “forbidden comparison,” arises when treated units are incorrectly used as controls in the conventional methodology. Essentially, this means that within the TWFE framework, some units that have already received treatment are mistakenly considered as part of the control group. Such a methodological approach can lead to significant biases in the estimation process, undermining the reliability of the results (Baker et al., 2022). To tackle this issue and address the potential bias, we adopted the estimators developed by Sun and Abraham (2021). Our model is shown as follows:


$$\begin{array}{l} y_{it}=\alpha_{i}+\lambda_{t}+\underset{g\notin C}{\sum }\underset{l\neq -1}{\sum }\mu_{g,l}\left(1\left\{E_{i}=g\right\}\cdot D_{it}^{l}\right)+{\;\varepsilon \;}_{it} \end{array}$$

where *y*_*it*_ denotes the hours of informal care provided and annual household income for individual *i* at time *t*. *E*_*i*_ is the time when individual *i* is initially covered by the LTCI, which is equal to “∞” for never-treated individuals. Whether an individual is covered by the LTCI or not can be determined by their eligibility rule (refer to *Methods* section for detailed information). *g* ∈{2012, 2015, 2017, ∞}, indicating disjoint cohorts. We set *C* = {∞} because there is a never-treated cohort. *l* denotes the relative time between *E*_*i*_ and *t*. $D_{it}^{l}$ is an indicator for individual *i* being *l* periods away from the initial treatment at time *t*. For the treatment groups, $D_{it}^{l}$ ∈{0, 1}, with $D_{it}^{l}$ = 1 if individual *i* is in *l* periods away from the initial treatment at time *t*, and $D_{it}^{l}$ = 0, otherwise. For never-treated individuals, $D_{it}^{l}$ = 0. The coefficient μ is the estimate for the cohort average treatment effects on the treated. α and λ control for the individual and year fixed effects, respectively. ε is a random error term. Standard errors are clustered at the city level to account for the possible correlation among the observations in the same city.

#### Propensity score matching

To deal with the endogeneity problem such as selection bias caused by the possibly nonrandom selection of LTCI pilot cities, we combined the DID approach with the PSM (Heckman et al., 1998). We used the logit model to estimate the propensity score for each individual by considering the following covariates: age, gender, residence area, education, health insurance types, number of living children, self-perceived health status, burden of care, household total income, and provincial GDP per capita. 1:6 caliper nearest neighbor matching is used and the caliper is set to 0.05. We did the balance tests with standardized differences instead of *t* tests, as the former is not affected by sample size and allows us to compare the relative balance of variables with different units (Austin, 2009). According to the results, the two groups were well balanced after matched with the differences generally less than 0.1, within an accepted level (Xu & Yang, 2021; Supplementary Table 4).

## Results

### Effects of the LTCI on Burdens Among Informal Carers

**Figure 1.**
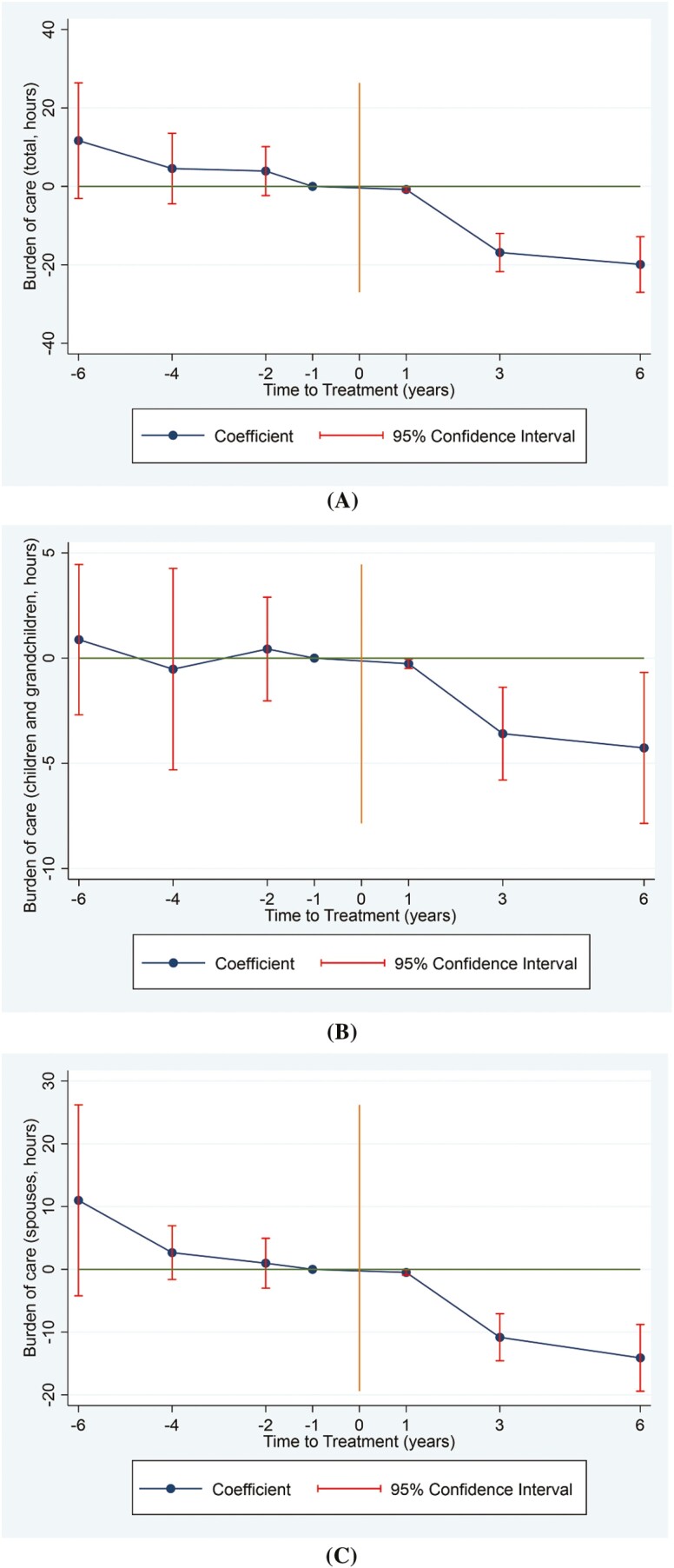
Effects of the long-term care insurance on total burden of care (A), burden of children/grandchildren (B), and burden of spouses (C) among informal carers. The year immediately preceding treatment is the reference point, with negative numbers denoting the pretreatment period and positive numbers the posttreatment period.

Table 1 and Figure 1 present the impact of LTCI on the burdens experienced by informal carers. As shown in column 1, LTCI had a significant and continuous effect, reducing the overall care burdens by 0.8, 16.9, and 19.9 hr per month in the first, third, and sixth years after the LTCI coverage, with an average reduction of 12.5 hr per month. Analyzing the specific sources of informal care, we observe that care provided by children/grandchildren and spouses experienced significant reductions of an average of 2.7 and 8.5 hr per month, respectively, as indicated in columns 2 and 3. These results suggest that LTCI alleviates care burdens for spouses of care recipients to a larger extent, and the impact is minor in the initial stage but gradually increases over time.

**Table 1.** Effects of the Long-Term Care Insurance on Burdens Among Informal Carers

Time to treatment, years	Burden of care, hours		
	Total (1)	Children and grandchildren (2)	Spouses (3)
Pre6	11.666	0.877	10.995
	(7.519)	(1.822)	(7.763)
Pre4	4.560	−0.525	2.663
	(4.589)	(2.441)	(2.184)
Pre2	3.920	0.432	0.981
	(3.183)	(1.258)	(2.027)
Post1	−0.785^***^	−0.265^**^	−0.488^**^
	(0.280)	(0.115)	(0.220)
Post3	−16.851^***^	−3.590^***^	−10.833^***^
	(2.482)	(1.125)	(1.911)
Post6	−19.896^***^	−4.264^**^	−14.104^***^
	(3.618)	(1.830)	(2.707)
ATT	−12.511^***^	−2.707^***^	−8.475^***^
	(1.877)	(0.923)	(1.374)
Covariates	Yes	Yes	Yes
*n*	7,186	7,070	7,070

*Notes*: Standard errors in parentheses. ATT represents average treatment effect for the treated group.

^**^ *p* < .05,

^***^ *p* < .01.

### Effects of the LTCI on Labor Market Participation

**Figure 2.**
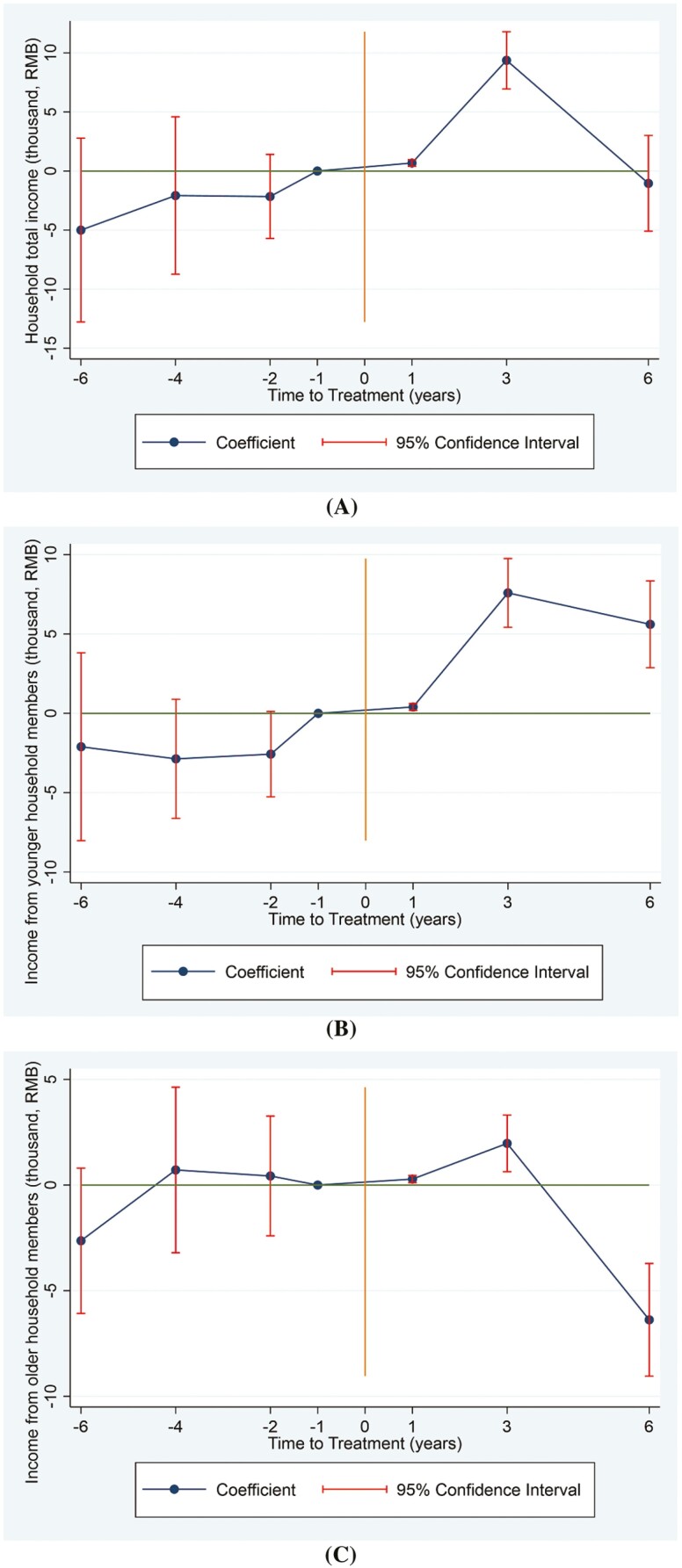
Effects of the long-term care insurance on household total income (A), income from younger household members (B), and income from older household members (C). The year immediately preceding treatment is the reference point, with negative numbers denoting the pretreatment period and positive numbers the posttreatment period.

Table 2 and Figure 2 present the impact of LTCI on labor market participation. As shown in column 1, LTCI increased household total income by 676 and 9,376 yuan per year in the first and third years after the LTCI coverage, with an average increase of 3,002 yuan per year. Column 2 shows that LTCI increased income from younger household members by 401, 7,590, and 5,609 yuan per year in the first, third, and sixth years after the treatment, with an average increase of 4,534 yuan per year. In column 3, income from older household members also slightly increased in the first and third years, which, however, decreased in the sixth year. This also contributes to the insignificant impact on household total income in the sixth year. This indicates that LTCI could promote the labor market participation of informal carers and improve their economic status, especially for the younger household members. It suggests that LTCI mainly affects the labor market participation of younger household members, as older people were less affected due to age and a lower likelihood of returning to the labor market. Our results indicate that LTCI allows younger household members to transition from informal care to paid work.

**Table 2.** Effects of the Long-Term Care Insurance on Labor Market Participation

Time to treatment, years	Labor market participation		
	Household total income (thousand, RMB) (1)	Income from younger household members (thousand, RMB) (2)	Income from older household members (thousand, RMB) (3)
Pre6	−5.002	−2.106	−2.636
	(3.972)	(3.021)	(1.754)
Pre4	−2.079	−2.870	0.715
	(3.399)	(1.914)	(1.999)
Pre2	−2.159	−2.572	0.429
	(1.816)	(1.371)	(1.446)
Post1	0.676^***^	0.401^***^	0.283^***^
	(0.140)	(0.109)	(0.087)
Post3	9.376^***^	7.590^***^	1.972^***^
	(1.237)	(1.106)	(0.685)
Post6	−1.045	5.609^***^	−6.377^***^
	(2.069)	(1.396)	(1.360)
ATT	3.002^***^	4.534^***^	−1.374^**^
	(0.913)	(0.743)	(0.575)
Covariates	Yes	Yes	Yes
*n*	7,184	7,184	7,184

*Notes*: Standard errors in parentheses. ATT represents average treatment effect for the treated group.

^**^ *p* < .05,

^***^ *p* < .01.

### Heterogeneous Effects of the LTCI Across the Health Insurance Types and Equivalent Income

We conducted further analysis to examine the heterogeneous effects of LTCI on the burden of care and labor market participation. Specifically, we first assessed the impact of LTCI on older people with different health insurance types. The main results are presented in Table 3(a). Columns 1 and 2 indicate that LTCI reduced the caregiving burdens among informal carers of the enrollees in the UEBMI and URRMI by 5.9 and 13.6 hr per month, respectively. It appears that the burdens on caregivers of enrollees in the URRMI were lightened to a larger degree. It is important to note that URRMI covers urban residents without formal employment and rural residents, whereas UEBMI covers urban residents with formal employment. Similar findings were obtained in our subgroup analysis focusing on income from younger household members. Specifically, we found that only households of care recipients enrolled in URRMI experienced significant impacts from LTCI, with increases of 4,282 yuan per year.

**Table 3.** Heterogeneous Effects of the Long-Term Care Insurance

Treatment effect	(a) By the health insurance types								(b) By the equivalent income		
	Burden of care (hours)		Household total income (thousand, RMB)		Income from younger household members (thousand, RMB)		Income from older household members (thousand, RMB)		Burden of care		
	UEBMI (1)	URRMI (2)	UEBMI (3)	URRMI (4)	UEBMI (5)	URRMI (6)	UEBMI (7)	URRMI (8)	Low income (1)	Middle income (2)	High income (3)
ATT	−5.872^***^ (2.180)	−13.578^***^ (2.705)	−0.488 (3.392)	1.930 (1.245)	−0.094 (2.260)	4.282^***^ (1.019)	−0.281 (3.391)	−2.040^***^ (0.670)	−34.690^***^ (6.415)	−4.416 (4.459)	−1.567 (2.891)
Covariates	Yes	Yes	Yes	Yes	Yes	Yes	Yes	Yes	Yes	Yes	Yes
*n*	1,522	4,105	1,522	4,103	1,522	4,103	1,522	4,103	1,211	1,374	1,544

*Notes*: UEBMI = Urban Employee Basic Medical Insurance; URRMI = Urban and Rural Resident Medical Insurance. Standard errors in parentheses. ATT represents average treatment effect for the treated group.

^**^ *p* < .05,

^***^ *p* < .01.

Additionally, we analyzed the heterogeneity of the effects on the burdens of informal care by economic status. Table 3(b) shows that LTCI significantly reduced burdens among informal carers with low income (by 34.7 hr per month), whereas no significant effects were observed among households with middle and high income.

### Robustness Checks

We conducted robustness checks to further validate our findings. Firstly, we performed a placebo test by creating a fake treatment group based on the new LTCI pilot cities introduced in 2020 and their inclusion in CHARLS. Specifically, we selected observations from six cities that were part of the new LTCI pilot program and sampled by CHARLS, assuming these cities received treatment in 2017. Upon analysis, we found no significant changes in both the burden of care and labor market participation within this fake treatment group (Supplementary Table 5). Secondly, given the small number of treated and control cities involved in our study, we implemented a cluster bootstrap procedure to enhance the robustness of the findings (Cameron & Miller, 2015). Encouragingly, the outcomes remained consistent with our initial findings (Supplementary Table 6). These robustness checks provide additional evidence that supports the robustness and reliability of our primary results. They demonstrate that our findings are not driven by specific treatment groups or potential clustering effects.

## Discussion

Understanding whether and how the introduction of the LTCI affects informal carers’ burdens and labor market participation can provide significant evidence for relevant policymaking, especially concerning residents’ welfare, regional economy, and population equity in LMICs. Taking China as a case study, this article offers compelling findings on the spillover effect of the LTCI on informal carers. Our results show consistent findings with existing studies that the LTCI significantly reduced the burdens among informal carers (Ma et al., 2019; Stabile et al., 2006). This is probably because the LTCI reduces the cost of receiving formal care, enabling more households to substitute formal care for informal care (Ettner, 1994). Furthermore, our analysis reveals that though the LTCI could significantly relieve the care burden for both the care recipients’ spouses and children/grandchildren, the spouses benefit more. This is likely because spouses, as primary caregivers for their partners, often shoulder more care burden initially before the introduction of LTCI. Then the absolute reduction of care hours would tend to be larger among spouses after the family receives support from LTCI (Ettner, 1994).

According to our results, we found that the introduction of the LTCI led to the increase in the household income. This finding indicates that the LTCI can promote the labor market participation of informal carers and potential informal carers, defined as the household members who have not yet provided informal care at the time being, which is in line with previous studies on the spillover effect on informal carers (Sugawara & Nakamura, 2014; Tamiya et al., 2011). For potential informal carers, the introduction of the LTCI may reduce their hesitancy to enter the labor market full-time, as they anticipate a lower care burden in the future and are more confident in balancing work and caregiving responsibilities. More importantly, our analysis shows that the LTCI had a more significant and effective impact on promoting the labor market participation of younger caregivers, in line with existing research (Fu et al., 2017; Yu et al., 2021). Younger caregivers, being of working age, often possess stronger labor force attachments and may have weaker economic strength, making them more inclined to spend more time on paid work to improve their economic status and fulfill their self-worth after being relieved from caregiving responsibilities. In contrast, we found that income from older household members slightly increased after the introduction of LTCI for less than 3 years but declined after 6 years. This is possibly because within the first several years, the older caregivers are partially relieved from care responsibilities to labor markets, but with the aging and thorough understanding of the LTCI policy, they become unable or unwilling to return to the labor force market due to factors like retirement plans or reduced physical capacity because they become confident that LTCI can guarantee them the receipt of formal care with a subsidized price if encountering disabilities in the future.

Heterogeneous results indicate that the burdens among informal carers of the enrollees in the URRMI were lightened to a larger extent, and only the younger household members in the URRMI significantly earned more than before the introduction of the LTCI. According to the policy design, the enrollees in the URRMI are mainly informal-sector workers and farmers, whereas the enrollees in the UEBMI are normally formal-sector employees (The State Council, 2016). Most informal-sector workers and farmers have limited and unstable income with little social security (Xu & Pei, 2022) and getting access to formal care becomes difficult for them and they have to shoulder the informal care burdens when without the LTCI (Kemper, 1992). However, with the LTCI, they have the option to receive formal care at a lower cost. This, coupled with the lack of stable income and social security, can incentivize them to return to the labor market and earn more money. Consequently, they benefit more in terms of both care burdens and income. Furthermore, our findings demonstrate that only informal carers from households with low income benefit from the LTCI in terms of care burdens. The underlying logic for this observation may be similar to the heterogeneous results by health insurance types. All in all, the introduction of LTCI has played a crucial role in promoting equity among individuals with diverse socioeconomic backgrounds.

This study provides meaningful findings on the diverse effects of the LTCI on informal carers in terms of care burdens and labor market participation in LMICs with China as an example. Several policy implications can be drawn. First, policymakers should consider expanding the coverage of the LTCI to include or even prioritize the URRMI enrollees nationwide. Currently, among the first-round pilot cities, only nine cover both the enrollees in the URRMI and UEBMI, whereas the other six only cover the enrollees in the UEBMI, possibly due to limited financing pools. However, our findings indicate that burdens of informal care primarily exist among the enrollees in the URRMI, and the introduction of the LTCI predominantly benefits this group in terms of both care burdens and household income. Expanding coverage to or giving priority to the URRMI can maximize the utility brought by the LTCI policy and promote equality among enrollees with different health insurance.

Second, in the longer run, policymakers should consider making the LTCI mandatory for people with income lower than a certain threshold, taking inspiration from the LTCI policies in high-income countries such as Germany. Simultaneously, safety nets for the poor should be developed to support this group of people with lower income. They are more likely to encounter barriers in receiving formal care without the LTCI, and their household members may have to give up jobs to provide them unpaid informal care (Campbell et al., 2010; Geraedts et al., 2000). Implementing this measure can guarantee greater equality in the fundamental welfare of older adults with different incomes and activate the economy in the aging society by promoting the supply of the labor market (Yang, Chang, et al., 2021).

This study has the following limitations. First, we could not observe the impact of the LTCI on the agricultural labor force because agricultural income is not included in the household income due to data limitations. Second, the burden of informal care is based on the self-reported information, where recall bias and varied subjective understandings could not be avoided. Third, due to limitations in the available data, we were unable to directly determine whether informal carers had joined the job market. As an alternative, we constructed the “household total income” as a proxy variable to indicate the status of labor market participation. This approach, while providing indirect evidence, has its limitations. In our construction of the household total income variable, we made a conscious effort to exclude components like pensions, which are not directly related to labor market participation. However, it is important to acknowledge that income level may not be a precise measure of labor market participation. Specifically, a higher income does not necessarily equate to full-time employment, nor does a lower income definitively indicate part-time employment.

## Conclusion

Our research offers compelling evidence that the implementation of LTCI has significantly alleviated the burdens faced by informal carers while boosting labor market participation, especially among younger members of households. Furthermore, our findings highlight that LTCI is particularly beneficial for informal carers of older individuals with lower incomes, as well as those from farming backgrounds or engaged in informal work sectors. These outcomes emphasize the crucial role of government initiatives in extending LTCI coverage. There is a pronounced need to either include or give priority to enrollees of the URRMI nationwide. Additionally, our study advocates for the mandatory application of LTCI for individuals with incomes below a specific threshold in the long term. This strategic approach could yield significant socioeconomic benefits, particularly for the most vulnerable segments of the population.

## Supplementary Material

gbae023_suppl_Supplementary_Table_S1-S6

## Data Availability

The data sets generated and/or analyzed during the current study are available in the China Health and Retirement Longitudinal Study (CHARLS) repository. http://charls.pku.edu.cn/en/.
