# Supplementary material for: Examining the Impact of Long-Term Care Insurance on the Care Burden and Labor Market Participation of Informal Carers: A Quasi-Experimental Study in China
Source: J Gerontol B Psychol Sci Soc Sci. 2024 Feb 23;79(5):gbae023. doi: 10.1093/geronb/gbae023 (PMC11227048; doi:10.1093/geronb/gbae023)
Supplement: gbae023_suppl_Supplementary_Table_S1-S6 [file gbae023_suppl_supplementary_table_s1-s6.docx]

*The Journals of Gerontology, Series B: Psychological Sciences and Social Sciences* Supplementary Material: Xingtong Pei, Wei Yang, & Mingming Xu. Examining the Impact of Long-Term Care Insurance on the Care Burden and Labor Market Participation of Informal Carers: A Quasi-Experimental Study in China.

Supplementary Table 1. Summary of the features of the LTCI

| Pilot cities | Eligibility | Premium | Benefits | Cognitive Impairment |
| --- | --- | --- | --- | --- |
| Qingdao | Enrolled in the UEBMI or the URRMI;  Disabled people assessed  as needing LTC, no age  restriction | Contributions by all residents to the health insurance scheme | The reimbursement rate is set at 90% for UEBMI enrollees and 70%-80% for URRMI enrollees. | Covered |
| Jingmen | Enrolled in the UEBMI or the URRMI;  Disabled people assessed  as needing LTC, no age  restriction | Contributions by all residents to the health insurance scheme | The reimbursement rate is linked to disability levels. 40%-80% for home care and 75% for institutional care. | Not covered |
| Shanghai | Enrolled in the UEBMI or the URRMI;  Disabled older people, aged 60 and above needing LTC | Contributions by all working-age and residents without retirement to the health insurance scheme | The reimbursement rate is set at 90% for home care and 85% for institutional care. | Covered |
| Chengdu | Enrolled in the UEBMI;  Disabled people assessed  as needing LTC, no age  restriction | Contributions by all residents to the health insurance scheme | The reimbursement rate is set at 75% for home care and 70% for institutional care. | Covered |
| Suzhou | Enrolled in the UEBMI or the URRMI;  Disabled people assessed  as needing LTC, no age  restriction | Contributions by all residents to the health insurance scheme | Fixed payment is linked to disability levels. 25-30 yuan/day/person for home care and 20-26 yuan/day/person for institutional care. | Not covered |
| Guangzhou | Enrolled in the UEBMI;  Disabled people assessed  as needing LTC, no age  restriction | Contributions by all residents to the health insurance scheme | The reimbursement rate is set at 90% for home care and 75% for institutional care. | Covered |
| Shangrao | Enrolled in the UEBMI;  Disabled people assessed  as needing LTC, no age  restriction | Contributions by all  working-age and retired  population, employers also  need to contribute. | LTC services include small subsidies for home care and institutional care. | Covered |
| Qiqihaer | Enrolled in the UEBMI;  Disabled people assessed  as needing LTC, no age  restriction | Contributions by all residents to the health insurance scheme | The reimbursement rate is set at 50% for home care and 55%-60% for institutional care. | Not covered |
| Chongqing | Enrolled in the UEBMI;  Disabled people assessed  as needing LTC, no age  restriction | Contributions by all residents to the health insurance scheme | Fixed payment is set at 50 yuan/day/person for home care and institutional care. | Not covered |
| Ningbo | Enrolled in the UEBMI;  Disabled people assessed  as needing LTC, no age  restriction | Appropriation from the accumulated balance of the UEBMI pooling fund | Fixed payment is set at 40 yuan/day/person for institutional care. | Not covered |

Notes. The table only displays the features of the pilot cities in the treatment group in this study. The LTCI policies presented are as of 2018.

Supplementary Table 2. List of the LTCI pilot cities

|  | Implementation date | Population coverage | Whether to be included in the study |
| --- | --- | --- | --- |
| Qingdao | 2012-7 | UEBMI | Yes |
|  | 2015-1 | URRMI |  |
| Changchun | 2015-5 | UEBMI; URBMI | No |
| Nantong | 2016-1 | UEBMI; URRMI | No |
| Anqing | 2017-1 | UEBMI | No |
| Jingmen | 2017-1 | UEBMI; URRMI | Yes |
| Shanghai | 2017-1 | UEBMI; URRMI | Yes |
| Shihezi | 2017-1 | UEBMI; URRMI | No |
| Chengdu | 2017-6 | UEBMI | Yes |
| Suzhou | 2017-6 | UEBMI; URRMI | Yes |
| Chengde | 2017-7 | UEBMI | No |
| Guangzhou | 2017-8 | UEBMI | Yes |
| Shangrao | 2017-9 | UEBMI | Yes |
| Qiqihaer | 2017-10 | UEBMI | Yes |
| Chongqing | 2017-12 | UEBMI | Yes |
| Ningbo | 2017-12 | UEBMI | Yes |

Notes. UEBMI, Urban Employee Basic Medical Insurance. URBMI, Urban Resident Basic Medical Insurance. URRMI, Urban and Rural Resident Basic Medical Insurance. Five pilot cities are not included in the study because there are no samples in CHARLS.

Supplementary Table 3. Descriptive statistics of 12,101 observations

|  | 2011, Mean (SD) | | 2013, Mean (SD) | | 2015, Mean (SD) | | 2018, Mean (SD) | | |  |
| --- | --- | --- | --- | --- | --- | --- | --- | --- | --- | --- |
|  | Control | Treatment | Control | Treatment | Control | Treatment | | Control | Treatment | |
| Burden of care, hours |  |  |  |  |  |  | |  |  | |
| Total | 20.60  (89.97) | 22.50  (104.00) | 6.72  (46.93) | 6.94  (48.04) | 9.49  (52.32) | 8.57  (55.47) | | 12.32  (67.25) | 11.26  (63.37) | |
| Children and grandchildren | 6.30  (51.60) | 3.63  (33.58) | 2.40  (22.06) | 1.36  (11.70) | 2.03  (18.33) | 2.64  (31.14) | | 3.56  (34.27) | 3.00  (31.94) | |
| Spouse | 11.60  (65.92) | 15.71  (91.23) | 2.62  (27.01) | 1.88  (29.97) | 5.15  (41.30) | 4.23  (36.29) | | 4.80  (43.82) | 5.37  (50.38) | |
| Household total income, thousand, RMB | 22.67  (34.61) | 23.06  (28.94) | 21.62  (31.93) | 22.24  (30.01) | 21.09  (31.54) | 23.09  (31.94) | | 26.25  (40.30) | 27.59  (40.46) | |
| Income from younger household members, thousand, RMB | 11.96  (23.28) | 14.03  (23.04) | 11.62  (23.87) | 12.29  (23.59) | 7.84  (19.09) | 9.43  (19.94) | | 9.56  (24.23) | 11.39  (29.77) | |
| Income from older household members, thousand, RMB | 10.58  (23.69) | 9.06  (16.08) | 9.96  (19.78) | 9.95  (16.50) | 13.25  (24.05) | 13.66  (21.09) | | 16.62  (31.15) | 16.00  (26.82) | |
| Age | 60.16  (10.43) | 60.11  (9.97) | 60.34  (10.26) | 60.30  (10.29) | 59.76  (9.92) | 59.53  (9.74) | | 60.65  (9.76) | 60.45  (9.62) | |
| Gender, % |  |  |  |  |  |  | |  |  | |
| Male | 50.59 | 48.60 | 47.90 | 48.04 | 48.03 | 47.36 | | 47.21 | 46.23 | |
| Female | 49.41 | 51.40 | 52.10 | 51.96 | 51.97 | 52.64 | | 52.79 | 53.77 | |
| Residence area, % |  |  |  |  |  |  | |  |  | |
| Rural | 40.90 | 36.34 | 24.75 | 24.88 | 28.37 | 27.68 | | 26.86 | 21.08 | |
| Urban | 59.10 | 63.66 | 75.25 | 75.12 | 71.63 | 72.32 | | 73.14 | 78.92 | |
| Marital status, % |  |  |  |  |  |  | |  |  | |
| Married | 86.08 | 86.45 | 85.93 | 86.23 | 87.46 | 87.68 | | 86.21 | 85.99 | |
| Single | 13.92 | 13.55 | 14.07 | 13.77 | 12.54 | 12.32 | | 13.79 | 14.01 | |
| No. of children | 2.51  (1.78) | 2.47  (1.85) | 2.33  (1.39) | 2.23  (1.43) | 1.99  (1.24) | 1.94  (1.25) | | 2.18  (1.25) | 2.14  (1.29) | |
| No. of total household members | 3.31  (1.61) | 3.25  (1.54) | 2.57  (1.25) | 2.54  (1.13) | 2.40  (1.05) | 2.47  (1.09) | | 2.77  (1.45) | 2.64  (1.21) | |
| No. of younger household members | 1.47  (1.55) | 1.40  (1.44) | 0.76  (1.20) | 0.74  (1.05) | 0.62  (0.89) | 0.65  (0.85) | | 1.02  (1.39) | 0.86  (1.15) | |
| No. of older household members | 1.84  (0.47) | 1.85  (0.48) | 1.81  (0.40) | 1.81  (0.40) | 1.78  (0.51) | 1.82  (0.54) | | 1.75  (0.44) | 1.77  (0.42) | |
| Self-perceived health status | 3.36  (1.04) | 3.38  (1.06) | 3.31  (1.07) | 3.30  (1.03) | 3.30  (1.08) | 3.29  (1.04) | | 2.78  (1.02) | 2.77  (0.97) | |
| No. of chronic diseases | 1.79  (1.95) | 2.13  (2.20) | 1.71  (1.66) | 1.59  (1.65) | 1.34  (1.48) | 1.21  (1.41) | | 2.10  (1.91) | 2.00  (1.80) | |
| No. of ADL limitations | 0.14  (0.72) | 0.10  (0.64) | 0.10  (0.54) | 0.09  (0.55) | 0.05  (0.35) | 0.06  (0.33) | | 0.05  (0.34) | 0.06  (0.41) | |
| No. of IADL limitations | 0.30  (0.96) | 0.21  (0.81) | 0.25  (0.85) | 0.20  (0.75) | 0.13  (0.53) | 0.11  (0.50) | | 0.17  (0.63) | 0.19  (0.69) | |
| Level of education, % |  |  |  |  |  |  | |  |  | |
| No formal education | 40.07 | 37.42 | 35.64 | 36.15 | 33.26 | 34.40 | | 33.88 | 34.04 | |
| Elementary or middle school | 40.23 | 43.66 | 43.61 | 45.70 | 50.25 | 50.72 | | 47.74 | 50.15 | |
| > = high school | 19.70 | 18.92 | 20.75 | 18.15 | 16.49 | 14.88 | | 18.37 | 15.81 | |
| Health insurance types, % |  |  |  |  |  |  | |  |  | |
| No health insurance | 12.89 | 12.47 | 6.56 | 6.89 | 10.28 | 9.28 | | 2.97 | 3.46 | |
| UEBMI | 17.79 | 17.85 | 26.48 | 20.34 | 26.52 | 24.16 | | 26.72 | 24.85 | |
| URRMI | 64.00 | 66.45 | 63.14 | 70.11 | 62.42 | 66.88 | | 66.15 | 67.62 | |
| Others | 6.86 | 5.81 | 6.95 | 5.95 | 11.84 | 12.64 | | 12.07 | 12.95 | |
| Equivalent income, thousand, RMB | 123.30  (1494) | 225.10  (2192) | 25.19  (23.25) | 24.53  (21.28) | 16.51  (20.79) | 18.29  (21.14) | | 33.04  (29.02) | 33.61  (30.07) | |
| n | 1,939 | 465 | 2,545 | 639 | 2,432 | 625 | | 2,792 | 664 | |

Notes. Household total income represents the household income of household members. Income from younger household members represents the household income of household members excluding the main respondents, their spouses, and their parents. Income from older household members represents the household income of the main respondents, their spouses, and their parents.

Supplementary Table 4. Summary of standardized difference between the treatment and control group before and after matching

|  | 2011 | | 2013 | | 2015 | | | 2018 | |
| --- | --- | --- | --- | --- | --- | --- | --- | --- | --- |
|  | Raw | Matched | Raw | Matched | | Raw | Matched | Raw | Matched |
| Age | 0.078 | -0.001 | 0.012 | -0.006 | | -0.061 | -0.023 | -0.142 | -0.003 |
| Gender | -0.002 | -0.037 | -0.006 | -0.013 | | -0.035 | 0 | -0.026 | -0.004 |
| Regional type | 0.942 | -0.027 | 1.181 | -0.148 | | 1.094 | -0.114 | 1.232 | -0.002 |
| Education (ref: No formal education) |  |  |  |  | |  |  |  |  |
| < = middle school | 0.01 | 0.046 | 0.053 | 0.015 | | 0.099 | -0.013 | 0.114 | 0.03 |
| > = high school | 0.186 | -0.025 | 0.156 | -0.075 | | 0.081 | -0.05 | 0.104 | -0.071 |
| Health insurance type (ref: No health insurance) |  |  |  |  | |  |  |  |  |
| UEBMI | 0.193 | -0.036 | 0.21 | -0.147 | | 0.296 | -0.087 | 0.286 | -0.05 |
| URRMI/URBMI | 0.546 | 0.145 | 0.586 | 0.136 | | 0.483 | 0.046 | 0.329 | -0.05 |
| NCMS | -0.686 | 0.012 | -0.71 | 0.044 | | -0.617 | 0.04 | -0.576 | 0.083 |
| Others | 0.03 | -0.047 | 0.048 | -0.08 | | 0.159 | -0.004 | 0.236 | -0.004 |
| No. of children | -0.138 | -0.002 | -0.346 | 0.021 | | -0.501 | 0.01 | -0.493 | 0.018 |
| Self-perceived health status | 0.135 | 0.046 | -0.181 | 0.02 | | -0.14 | -0.005 | -0.231 | 0.007 |
| Burden of care | 0.073 | 0 | -0.028 | -0.028 | | -0.145 | -0.008 | -0.155 | -0.004 |
| Household total income | 0.259 | -0.036 | 0.257 | -0.004 | | 0.383 | 0.063 | 0.298 | 0.003 |
| Provincial GDP per capita | 0.978 | -0.032 | 0.74 | 0.078 | | 0.823 | 0.084 | 0.11 | -0.019 |

Notes. The results presented are derived from caliper nearest neighbor matching. The two groups are considered well balanced if standardized differences being less than 0.1.

Supplementary Table 5. Robustness checks: regression of the fake treatment group

|  | Burden of care  (1) | Household total income  (2) | Income from younger household members  (3) |
| --- | --- | --- | --- |
| ATT | -3.429  (6.126) | -1.012  (4.590) | -1.434  (1.834) |
| Covariates | Yes | Yes | Yes |
| n | 7,186 | 7,184 | 7,184 |

Notes. Standard errors in parentheses. ** p <0 .05, *** p <0 .01. ATT represents average treatment effect for the treated group.

Supplementary Table 6. Robustness checks: regression with the cluster bootstrap procedure

|  | Burden of care  (1) | Household total income  (2) | Income from younger household members  (3) | Income from older household members  (4) |
| --- | --- | --- | --- | --- |
| ATT | -10.0756**  (1.946) | 2.903***  (1.074) | 4.148***  (0.786) | -1.879***  (0.630) |
| Covariates | Yes | Yes | Yes | Yes |
| n | 14,073 | 14,073 | 14,073 | 14,073 |

Notes. Standard errors in parentheses. ** p <0 .05, *** p <0 .01. ATT represents average treatment effect for the treated group.
